# Proteomic Changes Resulting from Gene Copy Number Variations in Cancer Cells

**DOI:** 10.1371/journal.pgen.1001090

**Published:** 2010-09-02

**Authors:** Tamar Geiger, Juergen Cox, Matthias Mann

**Affiliations:** Department for Proteomics and Signal Transduction, Max-Planck Institute for Biochemistry, Martinsried, Germany; Stanford University School of Medicine, United States of America

## Abstract

Along the transformation process, cells accumulate DNA aberrations, including mutations, translocations, amplifications, and deletions. Despite numerous studies, the overall effects of amplifications and deletions on the end point of gene expression—the level of proteins—is generally unknown. Here we use large-scale and high-resolution proteomics combined with gene copy number analysis to investigate in a global manner to what extent these genomic changes have a proteomic output and therefore the ability to affect cellular transformation. We accurately measure expression levels of 6,735 proteins and directly compare them to the gene copy number. We find that the average effect of these alterations on the protein expression is only a few percent. Nevertheless, by using a novel algorithm, we find the combined impact that many of these regional chromosomal aberrations have at the protein level. We show that proteins encoded by amplified oncogenes are often overexpressed, while adjacent amplified genes, which presumably do not promote growth and survival, are attenuated. Furthermore, regulation of biological processes and molecular complexes is independent of general copy number changes. By connecting the primary genome alteration to their proteomic consequences, this approach helps to interpret the data from large-scale cancer genomics efforts.

## Author Summary

In the course of cancer development, cells lose regulation of the cell cycle and quality control of DNA replication. As a result, many genomic alterations accumulate, among them amplifications and deletions of chromosomal regions of varying sizes. Oncogenes that drive transformation often reside in amplified regions, while tumor suppressors are deleted, yet for thousands of genes the effect of altering gene copy number is unknown. Since only genomic alterations that ultimately affect protein levels can have functional importance, a global proteomic approach that directly measures such changes is desirable. Here, we examined output of chromosomal alterations on the proteins in a system-wide manner. We analyzed the global protein expression of cancer cells compared to normal cells using mass-spectrometry–based quantitative proteomics and quantified a large part of the expressed proteome. We compared the protein data to genomic data and matched changes in gene copy number to protein expression level changes for each gene. Overall, gene copy number changes explain only a few percent of observed protein expression changes. Knowledge of when genomic and proteomic changes correlate may help in a better understanding of regulatory mechanisms in tumor development.

## Introduction

Chromosomal aberrations are a hallmark of cancer cells. During transformation cells lose cell-cycle control and fidelity of DNA replication causing multiple changes in DNA copy numbers [1], [2]. Although chromosomal aberrations are associated with transformation, changes in DNA copy number can cause growth defects rather than cell growth [3], [4]. Therefore transformation requires specific genomic changes that enable tolerance to genomic instability and promote growth and survival. The identity of these specific altered genes that enable transformation is still unknown, and great efforts are made to achieve a better understanding of these gene changes and their effects. Technological developments in recent years have allowed high resolution genomic analysis using SNP arrays, and large scale projects have mapped the gene copy number changes in thousands of tumor samples [5], [6]. Another major step necessary for the interpretation of the biological significance of such studies that is missing so far is the analysis of the consequences of these alterations: to what extent they affect protein expression. This in turn would allow investigation and interpretation of potential biological function. Several studies have shown high correlation between the amplifications and deletions and changes in mRNA levels and were therefore able to predict amplifications and deletions based on global transcript measurements [7]–[11]. Still, only a few amplifications were associated with oncogenes, and some deletions with tumor suppressors, while the majority of these alterations could not be associated with known tumor promoting activities [5], [6]. Furthermore, the effects of co-amplifications and deletions of genes in the same regions as known tumor-related genes, are yet to be discovered. A priori it would be possible that proteins encoded in a given amplicon are uniformly overexpressed in accordance with genome copy number or alternatively, that the expression levels only of selected or none of the proteins changes. These different scenarios have very different implications when trying to assess potential biological and oncological effects of a given amplicon detected in a somatic cancer genome.

For better understanding of the general output of chromosomal changes, the protein level therefore has to be globally examined. Such knowledge can be crucial as it can suggest novel potential drivers of transformation and, as already shown in specific cases in the past, help determine treatment modalities and prognosis [12], [13]. To compare proteomic to genomic alterations in a system-wide manner deep coverage of the proteome is essential as it maximizes the chance to detect and accurately quantify the proteins expressed from amplified or deleted regions. Stable Isotope Labeling by Amino Acids in Cell Culture (SILAC) is an accurate method for quantitative mass-spectrometry based proteomics [14], [15]. Recent advances in SILAC-based proteomics using high resolution mass spectrometry [16], [17] enabled accurate proteome coverage of the complete yeast proteome [18] and large proportions of the mammalian proteome [19]. Based on these developments, we could now compare cancer cell lines containing multiple chromosomal alterations and normal diploid epithelial cells, and further compare these changes to genomic alterations detected by SNP arrays. This accurate analysis enabled us to find the output of thousands of genes with varying gene dosage, and thereby estimate their regulation and their potential impact.

## Results/Discussion

### In-depth proteomic analysis of breast cancer cells and comparison to SNP array copy number data

**Figure 1:**
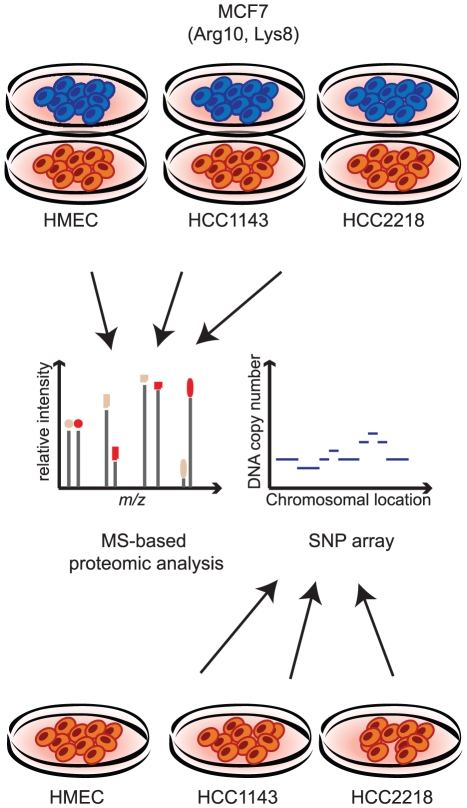
Measuring proteome and genome changes in cancer versus normal cells. For proteomic analysis lysates of each of the non-labeled cells (HMEC, HCC1143 and HCC2218) were mixed with lysate of SILAC-labeled MCF7 cells. Proteins were trypsin-digested and analyzed by LC-MS using high resolution mass spectrometry. For genomic analysis, genomic DNA was isolated from HMEC, HCC1143 and HCC2218 cells and hybridized with a SNP arrays.

To study the effects of genomic alterations on the protein level, we performed quantitative proteomic analysis of two aneuploid breast cancer cell lines and normal diploid cells. We SILAC-labeled the MCF7 breast cancer cell line with heavy lysine and arginine to serve as internal standard for quantification. The lysate of the labeled cells was combined with normal mammary epithelial cells (HMEC) or with two breast cancer cell lines - HCC2218, derived from a patient with Stage III ductal carcinoma and HCC1143, derived from a patient with Stage II ductal carcinoma (Figure 1). We analyzed each proteome mixture by enzymatic digestion and isoelelectric focusing of the resulting peptides followed by online liquid chromatography mass spectrometry on hybrid linear ion trap Orbitrap mass spectrometers. In total we identified and quantified 72,239 SILAC peptide pairs at 99% confidence. Quantification of cancer cell lines against normal cells was computed as the ‘ratio-of-ratios’ of each proteome against the internal MCF7 SILAC standard, requiring at least two quantification events per protein in each experiment. From biological triplicates, we identified and quantified 6,735 proteins (an average of more than 5,000 quantified proteins per cell line).

For the analysis of chromosomal aberrations, we mapped the copy number changes in the genome of HCC2218, HCC1143 and HMEC with SNP arrays (Affymetrix- Genome-wide Human SNP Array 6.0; Figure 1). Similar to the proteome analysis, we calculated the ratios of the signal in the cancer cell lines compared to the diploid control cells, then matched the chromosomal position with the gene, and determined the change in copy number as the median of the signals of all the probes annotated to the same gene. We matched between the proteins and the genomic data based on the gene name, enabling direct comparison of the level of almost every identified protein and its encoding gene.

**Figure 2:**
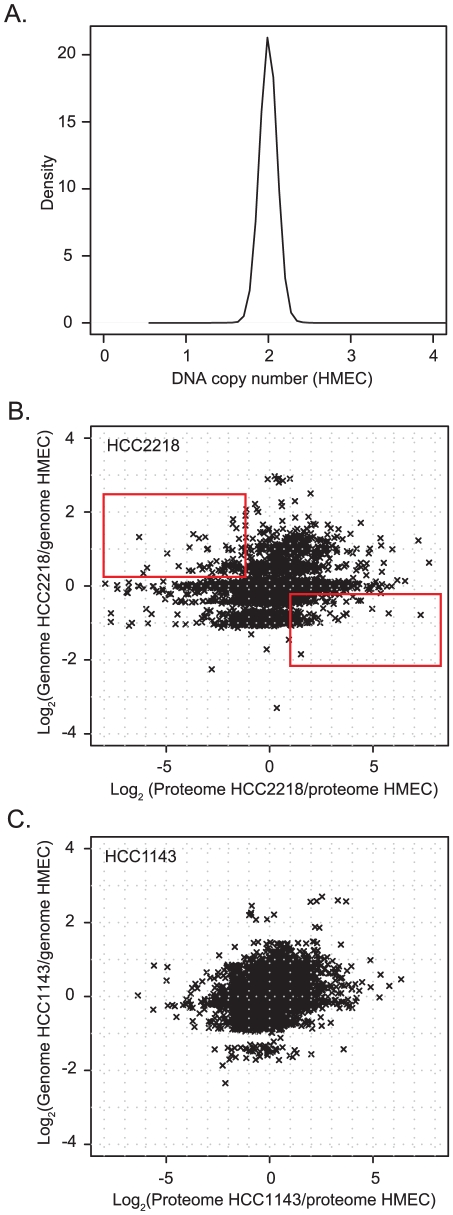
Comparison of gene copy number change to protein change. (A) Density plot of Affymetrix smooth signal in the HMEC control cells. A small peak at zero was removed, which was caused by probes for the Y-chromosome, which was absent in this female cell line. (B,C). Scatter plot of gene copy number in HCC2218 cells (B) or HCC1143 cells (C) normalized to the copy number of genes in HMEC vs. the ratio of the proteins in HCC1143 or HCC2218 cells relative to HMEC. The rectangle in the upper left part of (B) encloses genes with increased gene copy compared to control cells but decreased protein expression. The rectangle in the lower right contains single copy genes with increased protein expression compared to control cells.

A density plot of gene copy number of the HMEC indicates that these cells are diploid and therefore can serve as a normal control (Figure 2A). We normalized the proteomic and the genomic data of HCC2218 and HCC1143 cells to the control cells. Overall correlation between the change in gene copy number and the change in protein level determined in this way was low (0.22 for HCC2218 and 0.28 for HCC1143 cells). Only 4.8% and 7.8% of the protein level changes were determined by the copy number changes of the genes, significantly less than the percentage of transcriptome changes explainable by genome differences [9], [10], [20], [21]. This suggests that there is a tighter coupling between gene copy numbers and transcript changes than between gene copy numbers and protein level changes. The remaining changes of protein levels are presumably caused by other mechanisms of regulation of protein expression.

The plots of the gene copy number vs. the protein level show that the genome is distributed around integer values corresponding to 0, 1, 2, 3, 4 gene copies (Figure 2B and 2C). The distribution of the proteins encompassed many higher fold changes and was much less structured. Interestingly, many genes with higher than diploid copy number nevertheless have reduced protein expression and for many genes loss of one copy still resulted in increased protein expression compared to normal cells (rectangles in Figure 2B).

### Prediction of chromosomal aberration based on proteomic data

Many chromosomal changes can be inferred from mRNA data [7]–[9]. Given the depth and accuracy of our proteome measurement, we wanted to see whether despite the low overall correlation, gene amplifications and deletions can also be directly inferred from proteomic data and to find region-related proteomic changes. We developed a genome profiling algorithm that examines the correlation between the expression levels of proteins that are adjacent in a given chromosomal location. This algorithm orders proteins on each chromosome and checks for significant regional deviations of their log ratios from zero. For that purpose windows encompassing various numbers of adjacent proteins are moved along the chromosome, and the deviation of the window mean from zero is tested by one-sample t-test. A p-value is determined for each window size ranging from 3 proteins to the whole chromosome. The final amplification or deletion profile is then calculated from the window medians of all windows in which the average value differs significantly from zero. At each position each intersecting significant window is considered and among those the value that deviates most from zero is chosen. This value is reported in the amplification/deletion profile at this position.

**Figure 3:**
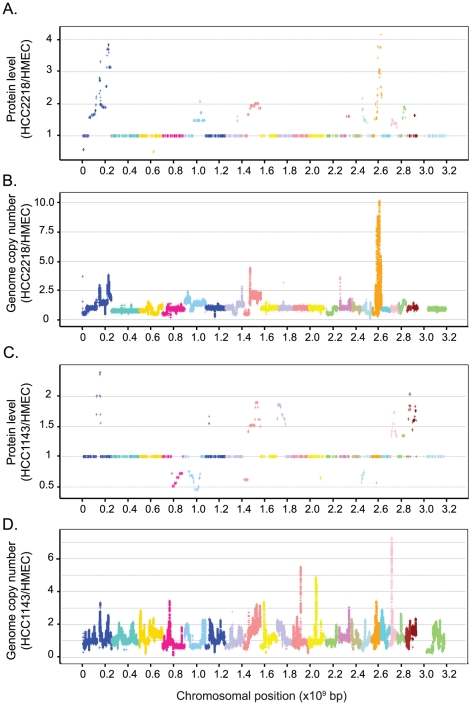
Genome profiling of genomic and proteomic data. Protein ratios were averaged according to their localization using the genome profiling algorithm (Materials and Methods). Calculated ratios of proteins in HCC2218 (A) or HCC1143 (C) versus HMEC are plotted against their chromosomal location. Smoothed data of gene copy number in HCC2218 (B) or HCC1143 (D) normalized to the control cells are plotted against their chromosomal location. Each color represents a different chromosome.

After genome profiling, the correlation between the calculated change in protein amounts at each genome position and the corresponding change in gene copy number was greatly increased (0.64 and 0.59 for HCC2218 and HCC1143, respectively). We plotted the calculated proteomic values against their chromosomal location to visualize amplifications and deletions along the chromosomes (Figure 3A and 3C). The genome profiling algorithm predicted and localized numerous aberrations. In HCC2218 cells we found very high level amplification in chromosomes 1 and 17, and lower amplifications in chromosomes 5, 7, 8, 14, 16, 19, 20, 21. We found only two small deletions in chromosomes 1 and 3. In HCC1143 cells we predicted amplifications in chromosomes 1, 6, 8, 10, 19, 20, 21 and 22, and deletions in chromosomes 4, 5, 8, 12, 16 and 17.

To examine whether our predictions were correct despite the low correlation between genome and proteome, we performed a similar alignment of the genomic data. We plotted the smoothed data of the SNP array (normalized to the control cells) directly according to the genomic location. Although not all aberrations had a detected proteomic output; remarkably, in each of the predicted locations, we indeed found a matching change in the SNP array data (Figure 3B and 3D). Thus accurate proteome measurements can indeed detect genome copy number changes, via the regional effects on protein expression level changes. Furthermore, these predicted changes agree with well known breast cancer genomic alterations, such as gains in chromosome 1q, 8q, 16p, 17q, 20q and losses in chromosomes 4q, 8p [22], [23].

### Functionality of genomic alterations

**Figure 4:**
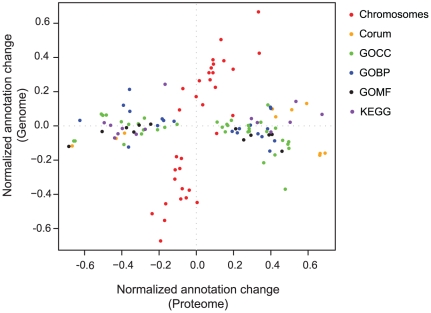
2D annotation distribution. Scatter plot of normalized annotation changes on the genome level against the protein level. Calculation of significance is detailed in the Materials and Methods section. The annotations analyzed were: chromosomes (red), Corum complex database (orange), gene ontology cellular component (GOCC; green), gene ontology biological processes (GOBP; blue), gene ontology molecular function (GOMF; black) and KEGG pathways (purple).

While the correlation between the gene copy number and the proteins was very low, it was still possible that the altered genes would globally affect specific pathways and processes, to confer a growth advantage to the aneuploid cells. We comprehensively analyzed each process to determine to what extent it is regulated on the protein level or on the genomic level. We developed a two-dimensional annotation distribution analysis tool (see Materials and Methods), to determine protein categories with significant co-regulation in the combined space of gene copy number and protein changes. We examined gene-ontology (GO) categories, KEGG pathways, protein complexes annotated in the CORUM database and distribution of genes to chromosomes (Figure 4). The only categories changing at the genome level were the chromosomes themselves and, as shown above, they only have a small overall effect on the proteome level. Almost all other statistically significant categories, including GO, KEGG and CORUM are distributed horizontally along the proteome direction, indicating that they cannot be directly attributed to broad changes in gene dosage (Table S1). As an example, Figure S1 illustrates the changes in oxidative phosphorylation genes and proteins in HCC2218. There was a clear increase in the abundance of proteins involved in this process, while most of the corresponding gene copy numbers were constant (Figure S1). Moreover, there were genes whose copy number changed, but the encoded proteins did not change accordingly. For example NDUFB9, ATP6V1H and ATP1C1, were amplified, and a single copy of ATP6V1B2 was deleted, but the protein levels stayed constant. In this case, clearly the copy number of genes belonging to this process had no effect on its functionality.

### Stable protein complexes maintain constant protein expression despite changed gene copy number and mRNA expression

**Figure 5:**
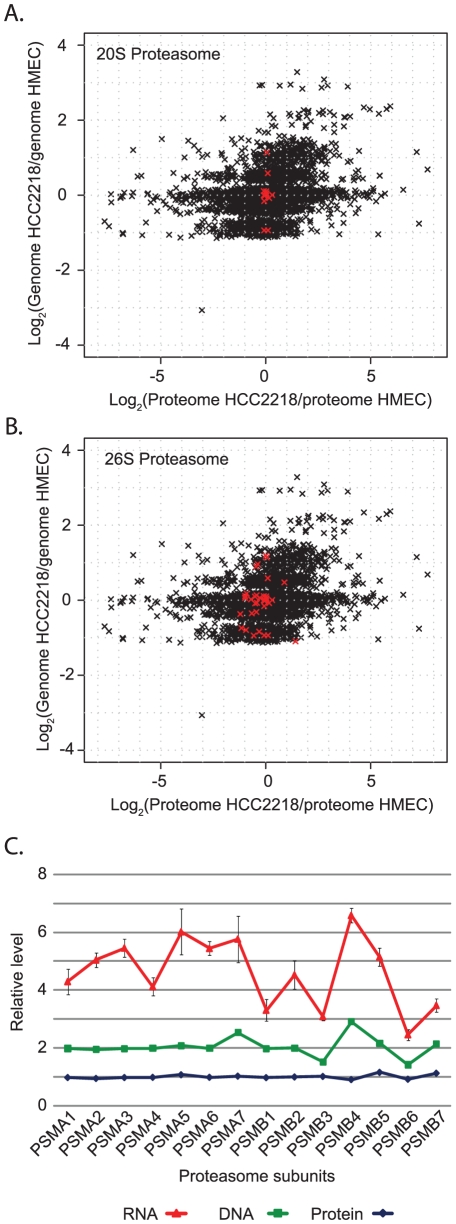
Distribution of proteasomal genes, proteins and mRNA. (A) Scatter plot of global ratio distribution of genes vs. proteins in HCC2218. The core 20S proteasome components are highlighted in red. (B) Scatter plot with the 26S proteasome highlighted in red. (C) Stacked plot of protein, gene and mRNA level of 14 proteasomal subunits, normalized to the level in HMEC.

Our two-dimensional annotation analysis further highlighted a number of protein complexes, such as the proteasome, ribosome, spliceosome and NADH dehydrogenase complex. We found that the proteins of these complexes always maintain equal protein ratios, despite variation in the gene copy number of their subunits (Figure 5A and Figure S2). Interestingly, this is strictly true for the core complexes components, but to a lesser degree for peripheral proteins, which can also be involved in other processes. The 20S proteasome, which includes seven alpha and seven beta subunits, is completely insensitive to gene dosage while the levels of the proteins from the whole 26S proteasome vary slightly (Figure 5A and 5B). Similarly, we found much higher variation in the spliceosome complex than in the 17S U2 snRNP subcomplex (Figure S2B). We further examined whether the determination of the exact ratios of the proteins in a core complex is due to regulation already at the level of mRNA and can be attributed to regulation of transcription or mRNA stability, or on the protein level and could be related to protein translation or degradation. We measured the mRNA levels of the proteasome core complex (seven alpha subunits and seven beta subunits) by real-time-PCR in HCC2218 cells. In contrast to the equal protein amounts, we found large variability in the mRNA levels of the subunits (Figure 5C). The correlation between mRNA and genes was 0.6, while the correlation between proteins and their corresponding genes was −0.1. Therefore, the main regulation of the protein amounts for this complex occurs at the protein level, rather than at the mRNA level. In accordance with these results, it has been shown that ribosomal subunits are synthesized in excess and those subunits that do not assemble into the complex are degraded [24]. Our results suggest that this mechanism occurs in many molecular complexes. For these complexes the abundance of the subunits is regulated by the amount of the whole complex, and this regulation is done only on the protein level.

### Oncogenes are found as amplified genes encoding overexpressed proteins

We showed above that cellular processes and molecular machines do not obey gene dosage changes. But as primary events in transformation, amplification of deletion of key regulatory genes may impact the functionality of the whole process. Indeed, oncogenes and tumor suppressors are often amplified or deleted in the genome [5]. For such aberrations to affect transformation, the gene copy number change must positively correlate with a protein level change. For example, HCC2218 cells have a known amplification of the ERBB2 gene, and indeed our data show that the protein is >50 fold increased compared to HMEC. We searched whether more of the amplified or deleted genes with correlative protein level changes have known oncogenic or tumor suppressor activities by comparing our data to the Sanger institute ‘cancer gene census’ [25]. Among this list of genes that were amplified, deleted, mutated or translocated in various cancers, we selected those in which changes in genome copy number positively correlated with our measured proteome changes (Table S2). For instance, among the amplified genes we found AKT1 and CCND1 in HCC1143 cells and we found CDH1 to be deleted in HCC2218 cells.

**Figure 6:**
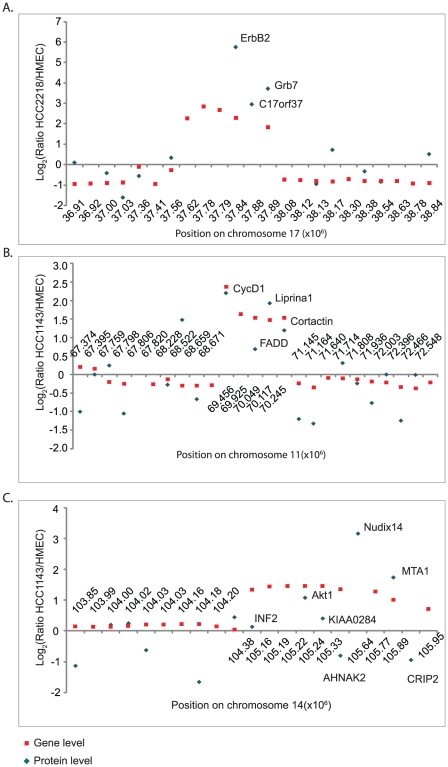
ERBB2, CCND1, and AKT1 amplicons. Zoom-in on the small amplicons surrounding ERBB2 in HCC2218 cells (A) CCND1 in HCC1143 cells (B) and AKT1 in HCC1143 (C). Fold changes in gene copy number compared to HMEC are marked with red rectangles; the fold changes in protein level are marked with blue diamonds.

We zoomed-in on the small amplicons encompassing ERBB2, CCND1 and AKT1 to examine the effects of these amplifications on the expression levels of adjacent genes (Figure 6). The ERBB2 amplicon is very well studied [26] and includes five genes; of these we quantified three proteins: ErbB2, C17orf37 and Grb7, all of which were highly over-expressed (Figure 6A). The significance of ErbB2 and the effects of its inhibition are well known [27], [28]. Its amplification is examined routinely in the clinic and predicts responsiveness to treatment with trastuzumab. Over-expression of Grb7, a mediator of receptor tyrosine kinase and integrin signaling, was also shown to correlate with tumor aggressiveness [29]. The function of the gene-product of C17orf37 is still unknown, but its protein overexpression along with ErbB2 and Grb7 makes it an interesting candidate for functional studies in breast cancer.

The amplicon surrounding CCND1 gene includes five genes – of them we quantified four (Figure 6B). CCND1 encodes the cell-cycle regulator Cyclin D1, whose overexpression is known to enhance tumor growth in multiple cancer types [30]–[32]. The same amplification event induced overexpression of Liprinα1 and Cortactin. Overexpression of Liprinα1 may promote cell migration [33], and Cortactin overexpression was reported to be associated with increased tumor aggressiveness [34]. In contrast, expression of Fas-Associated protein with Death Domain, FADD, was much lower than expected from the gene amplification. FADD is an adaptor protein that mediates signals from death receptors to caspase 8 during apoptosis [35]. Possibly, amplification-induced protein overexpression has deleterious results for cancer cells, which therefore control its overexpression.

The amplicon surrounding AKT1, an oncoprotein that mediates cell growth and survival [36], is located at the end of chromosome 14, and includes 11 genes. These contain NUDT14 and MTA1, which show even higher fold overexpression. MTA (metastasis-associated protein) is involved in chromatin remodeling, and its overexpression has been associated with a more aggressive phenotype of some tumors [37]. NUDT14 is a minimally characterized protein implicated in the regulation of carbohydrate metabolism [38]. The high expression of these genes suggests investigation of possible tumor-promoting role in these cells. In contrast, four other amplified genes were not overexpressed as proteins and some of them were even down-regulated. Crip2 and INF2 are actin binding proteins, suggesting a potential role in cell adhesion and migration [39], [40]. In agreement with the opposing changes of Crip2 gene and protein levels, the promoter of Crip2 was shown to be methylated in cancer cell lines and animal models [41], offering a possible mechanism to eliminate the effect of the amplification. The functions of AHNAK2 and KIAA0284 are still unknown. Downregulation of proteins encoded by amplified genes suggests that overexpression of these proteins may have negative effects on the cells.

Extrapolating from the proteins with a known role in the etiology of cancer, we created a list of potential novel regulators of transformation. We listed the overexpressed proteins encoded by amplified genes in HCC2218 and in HCC1143 cells (Table S3). These proteins were upregulated as a result of gene amplification, and their overexpression may have given a growth advantage to these cells. In contrast, reduced expression of amplified proteins may point to a negative effect on tumor growth. We performed similar analyses for the deleted regions, and listed the downregulated proteins, which may function as tumor suppressors, and the upregulated protein, which may be important proteins for cell growth. Functional research targeted towards these proteins could lead to identification of novel drivers of transformation and crucial regulatory proteins.

### Conclusions

We conclude that with high coverage of the proteome and high quantification accuracy, multiple chromosomal aberrations can be predicted directly from the proteomic data. Furthermore, proteomics can determine which genes in an amplified region are expressed at all and which are changing at the endpoint of the gene expression cascade – the level of the proteins. As expected, the expression of some oncogenes and tumor suppressors is affected by gene copy number. However, our data clearly show that in the majority of cases, there is no direct correspondence between the gene copy number change and the corresponding protein change. We suggest that proteomics is a useful complement to widely employed gene copy number analysis. It can determine if genome amplifications or deletions have a downstream effect on the level of the protein - a precondition for a potential impact on the transformation process.

## Materials and Methods

### Cell culture and SILAC labeling

Human mammary epithelial cells (HMEC) were obtained from Lonza and cultured in mammary epithelial cell growth medium (ECACC- Health Protection Agency). HCC1143 and HCC2218 cells were obtained from the American Type Culture Collection (ATCC), and grown in RPMI containing 10% FBS. MCF7 cells were obtained from the German Collection of Microorganisms and Cell Cultures (DSMZ). MCF7 cells were SILAC labeled by culturing them in DMEM where the natural lysine and arginine were replaced by heavy isotope labeled amino acids, L-^13^C_6_^15^N_4_-arginine (Arg10) and L-^13^C_6_^15^N_2_-lysine (Lys8). Labeled amino acids were purchased from Cambridge Isotope Laboratories, Inc, USA. The medium was supplemented with 10% dialyzed serum. Cells were cultured for approximately 8 doublings in the SILAC medium to reach complete labeling. For proteomic analysis each of the cell lines was analyzed in biological triplicates. The first two replicates were lysed with modified RIPA buffer (50 mM Tris HCl pH 7.4, 150 mM NaCl, 1 mM EDTA, 1% NP40, 0.25% sodium deoxycholate and protease inhibitors) at 4°C. Following lysis, lysates were centrifuged at 14,000 rpm at 4°C. Proteins were then precipitated over-night with acetone, and resuspended in 8 M urea (6 M urea, 2 M thiourea). Cells of the third replicate were lysed with a buffer containing 4% SDS, 100 mM Tris-HCl pH 7.6 and 100 mM DTT. Lysates were incubated at 95°C for 5 min, and then briefly sonicated.

### DNA isolation and SNP arrays

Genomic DNA was isolated from the cells using QIAmp DNA Blood Maxi Kit. DNA was hybridized with the Affymetrix Genome-Wide Human SNP Array 6.0 according to the manufacturer's instructions. SNP array analysis was done in the Microarray DNA facility at the Max Planck Institute of Molecular Cell Biology and Genetics, Dresden. Raw files were analyzed with “Copy Number and LOH analysis” algorithm from the Affymetrix Genotyping console. We used the default settings with the HapMap270 as reference, quality assessment and regional GC correction configuration. The ‘SmoothSignal’ column from the Affymetrix software output was used directly for the genome profile in Figure 3. For the comparison with the proteomic data, we determined the copy number of the gene as the median of the smoothed signal of the probes annotated with the corresponding gene name. These values were normalized to the gene copy number in the control cells, which are always diploid (Figure 2A).

### Trypsin digestion

Each of the non-labeled samples (HMEC, HCC1143 or HCC2218) was mixed at a ratio 1∶1 with labeled MCF7 cells. Two methods were used for trypsin digest. In-solution digestion was used for the first two replicates, where cells were lysed with RIPA buffer. Filter Aided Sample Preparation (FASP) [42] was used when lysis was done with SDS-based buffer. For in-solution digest, proteins were reduced by incubation with 1 mM DTT for 30 min at room-temperature, followed by alkylation with 55 mM iodoacetamide for 30 min at room-temperature in the dark. Next, proteins were digested with Lysyl Endopeptidase (LysC) at a concentration of 1∶50 (w/w) for three hours. Proteins were then diluted 4 fold in water, and digested with trypsin over-night at a concentration of 1∶50 (w/w). FASP digestion was performed as previously described [42]. Briefly, proteins were loaded on microcon-30 kDa filters. Following two washes with urea, proteins were alkylated with 50 mM iodoacetamide. Filters were washed twice with urea and twice with 40 mM ammonium bicarbonate, and digested over-night with Trypsin (1∶50; w/w) at 37°C. Peptides were desalted on Milli-SPE C18 extraction cartridges (Millipore).

### RT–PCR

mRNA was isolated from HMEC, HCC1143 and HCC2218 using PrepEase RNA Spin Kit (USB). Two micrograms of each mRNA were reverse-transcribed using First strand cDNA Synthesis Kit (Fermentas) with oligo-dT primers. For real-time PCR, we used IQ SYBR-green Supermix (Biorad) on a C1000 Thermal Cycler (Biorad). Method included 40 cycles of amplification with annealing and elongation temperature of 54°C or 58°C. Primers for GAPDH were used for normalization. List of primers is given below (5′-3′):

PSMA1:for CTGTTAAACAAGGTTCAGCCAC rev CCAAACACTCCTGACGCATA

PSMA2:for TGTTGGAATGGCAGTAGCAG rev TGCAGCCAAAAGGTCTAACA

PSMA3:for TGTTGGAATGGCAGTAGCAG rev TGCAGCCAAAAGGTCTAACA

PSMA4:for TCAATGAGGACATGGCTTGC rev AGGGACGTTTTCCTCCAAAT

PSMA5:for GCTCACATAGGTTGTGCCATG rev CTGGGGTCCTTTCTCATCAA

PSMA6:for GGCTATGAGATTCCTGTGGAC rev GAAGCTGGTTGACTCAGTTTGTT

PSMA7:for CTTTTGAGAGTCGCGGCGGA rev CCGCACTGTTCTTTCATCCTG

PSMB1:for AAGAAGGAAAGGGGGCTGTA rev TCTCTCTCAGCCGCAGAAAT

PSMB2:for GTGAGAGGGCAGTGGAACTC rev GTGAGAGGGCAGTGGAACTC

PSMB3:for CGGAATGTGTGAGTCCCTCT rev CTGGGAACAGGGTTAGTCCA

PSMB4:for GCCAGATGGTGATTGATGAG rev GGGCTTCATAGGCTACACCA

PSMB5:for ACTTCCCTTACGCAACATGG rev GCCTAGCAGGTATGGGTTGA

PSMB6:for GGCGGACTCCAGAACAACC rev CCAGTGGAGGCTCATTCAGT

PSMB7:for CTGTGTCGGTGTATGCGCCA rev GCAACAACCATCCCTTCAGT

GAPDH: for TGGTATCGTGGAAGGACTCATGAC rev ATGCCAGTGAGCTTCCCGTTCAGC

### Peptide fractionation

Peptides were separated according to their isoelectric-point using an Agilent 3100 OFFGEL fractionator (Agilent,G3100AA) as described previously[43]. Briefly, we used 13 cm IPG Drystrips, pH 3–10 (GE Healthcare). Strips were rehydrated for 20 min with a solution containing 5% glycerol and 1∶50 dilution of IPG buffer, pH 3–10 (20 µl/well). Peptides were diluted in 5% glycerol and IPG buffer. A total of 100 µg of peptides were loaded on each strip. Focusing was done for 20 kVh with a miximum current of 50 µA and power of 200 mW. Fractions were acidified by adding 1% TFA, 0.5% acetic acid and 3% acetonitrile. Prior to LC-MS analysis peptides were concentrated and desalted on StageTips[44].

### LC-MS analysis

Peptides were separated by reverse-phase chromatography on an in-house made 15 cm column (inner diameter 75 µm, 3 µm ReproSil-Pur C_18_-AQ media), using a nanoflow HPLC system (Proxeon Biosystems). HPLC was coupled on-line via a nanoelectrospray ion source (Proxeon Biosystems) to a LTQ-Orbitrap mass spectrometer (Thermo Fisher Scientific). Peptides were loaded onto the column with buffer A (0.5% acetic acid) with a flow rate of 500 nl/min, and eluted with 90 min linear gradient at a flow rate of 250 nl/min. After the linear gradient the column was washed with 90% buffer B and re-equilibrated with buffer A. Mass spectra were acquired in the positive ion mode applying a data-dependent automatic switch between survey scan and tandem mass spectra (MS/MS) acquisition. Samples were analyzed with a ‘top 5’ method, acquiring one Orbitrap survey scan in the mass range of m/z 300–2000 followed by MS/MS of the five most intense ions in the LTQ. The target value in the Orbitrap was 1,000,000 ions for survey scan at a resolution of 60,000 at m/z 400 using lock masses for recalibration[45]. Fragmentation in the LTQ was performed by collision-induced dissociation with a target value of 5,000 ions. Ion selection threshold was 1000 counts.

### MS data analysis

Raw MS files from the LTQ-Orbitrap were analyzed by MaxQuant[14], [46] (version 1.0.14.3). MS/MS spectra were searched against the decoy IPI-human database version 3.62 containing both forward and reverse protein sequences by the MASCOT search engine (version 2.2.04, Matrix Science). Parent mass and fragment ions were searched with maximal initial mass deviation of 7 ppm and 0.5 Th, respectively. The search included variable modifications of methionine oxidation and N-terminal acetylation, and fixed modification of cystein carbamidomethylation. Peptides of minimum 6 amino-acids and maximum of two missed cleavages were allowed for the analysis. For peptide and protein identification false discovery rate (FDR) was set to 0.01. In case the identified peptides of two proteins were shared by two proteins (homologs or isoforms), the two proteins were reported by MaxQuant as one protein group. Complete protein and peptides lists are given as Table S4 and Table S5.

### Genome profiling algorithm

The algorithm is applied to the log ratios between relative protein levels of a cancer cell to a normal cell. Chromosomal locations are assigned to proteins according to the Ensembl annotation that is included into Uniprot. On each chromosome the sequentially ordered proteins are checked for significant regional deviations of their normalized log ratios from zero. For that purpose windows encompassing various numbers of adjacent proteins are moved along the chromosome, and the deviation of the window mean from zero is tested with a one-sample t-test. Window sizes range from 3 proteins to the whole chromosome in steps of factors of square root of 2. Each log p-value was transformed in a window-length dependent way to a posterior error probability, applying Bayes rule to two-dimensional histograms. To correct for multiple hypothesis testing, a false discovery rate of 2% was applied by permutation-based estimation on the basis of 10 randomized genomes. The final amplification or deletion profile is then calculated from the window medians of all windows in which the average value differs statistically significantly from zero. At each position each intersecting significant window is considered and among those the value is taken that deviates most from zero. This is then the value of the amplification/deletion profile reported at this position. To obtain copy numbers, these values have to be exponentiated and multiplied by two. Protein ratios and the corresponding gene copy number changes are given in Table S6. Protein ratios after genome profiling are given in Table S7.

### Two-dimensional annotation analysis

Categorical annotation is supplied in form of Gene Ontology (GO) biological process (BP), molecular function (MF) and cellular component (CC) as well as participation in a KEGG pathway and membership in a protein complex as defined by CORUM. The chromosome of the corresponding gene was considered as an additional protein annotation. For each annotation term proteins are separated into two groups, one containing the proteins annotated with this term and the other containing the complement. A two-dimensional two-sample test then finds significant difference between the two-dimensional means of the two protein populations. Here, the two numerical dimensions consist of log protein ratio and log copy number ratio, but the algorithm would apply to other data types as well. The specific test we use is a two-dimensional version of the non-parametric Mann-Whitney test. Multiple hypothesis testing is controlled by using a Benjamini-Hochberg false discovery rate threshold of 5%. For categories that are significant a two-dimensional difference score is calculated by determining the average rank of the proteins belonging to the category. This average rank is then rescaled to the interval between −1 and 1. A value of 1 in one of the dimensions would mean that all members of this category are the largest values in this dimension, while a value of 0 means that the ranks of the members of the category are distributed in the same way as the background proteins, having no significant bias towards larger or smaller values.

## Supporting Information

Figure S1 Oxidative phosphorylation changes. Gene copy number changes (A) and proteomic changes (B) in oxidative phosphorylation proteins in HCC2218 cells. Oxidative phosphorylation proteins that were identified in HCC2218 were selected and network was established using the STRING database[47] (Version 8.2). Graphical network was done in Cytoscape (Version 2.6.3). Colors distinguish between highly up-regulated (>3 fold; red), up-regulated (1.6–3 fold; orange), constant (0.6–1.6 fold; yellow), down-regulated (<0.6; green), identified but not quantified (grey).
(2.93 MB EPS)

Figure S2 Protein complexes. Distribution of genes versus proteins. (A) Scatter plots of gene and protein distribution in HCC1143. Highlighted in red are the 26S or 20S proteasomal subunits. (B) Ratio distribution of genes and proteins in HCC1143 and HCC2218, with complexes highlighted in red.
(5.84 MB EPS)

Table S1 Changes of functional categories on the proteome and genome level.
(0.13 MB PDF)

Table S2 Cancer-associated genes that have a change in gene copy number.
(0.08 MB PDF)

Table S3 Amplified and deleted genes and matching changing proteins.
(0.16 MB XLS)

Table S4 Complete protein table.
(2.39 MB XLS)

Table S5 Peptide table.
(9.21 MB XLSX)

Table S6 Merged table of protein ratios and gene copy number.
(1.82 MB XLS)

Table S7 Genome profiled protein ratios.
(1.25 MB XLS)
