# Supplementary material for: Proteomic Changes Resulting from Gene Copy Number Variations in Cancer Cells
Source: PLoS Genet. 2010 Sep 2;6(9):e1001090. doi: 10.1371/journal.pgen.1001090 (PMC2932691; doi:10.1371/journal.pgen.1001090)
Supplement: Table S1 — Changes of functional categories on the proteome and genome level. (0.13 MB PDF) [file pgen.1001090.s003.pdf]

Table S1: Changes of functional categories on the proteome and genome level. Gene ontology (GO) biological processes (GOBP), molecular functions (GOMF), cellular compartments (GOCC), KEGG pathways, molecular complexes (Corum) and chromosomes were analyzed using two-dimensional annotation analysis algorithm (see Methods). Significant changes were determined with a maximum false discovery rate (FDR) of 0.05.

Cell	Type	Name	Proteome change	Genome change	FDR
HCC2218	Chromosome	5	0.1	0.5	0.0E+00
	Chromosome	3	-0.2	-0.5	0.0E+00
	Chromosome	2	-0.2	-0.7	0.0E+00
	Chromosome	1	0.1	0.3	2.1E-13
	Chromosome	X	-0.1	-0.4	4.4E-09
	Chromosome	4	-0.1	-0.4	4.7E-09
	Chromosome	16	-0.1	-0.4	1.9E-08
	Chromosome	20	0.1	0.4	2.8E-07
	Chromosome	13	-0.2	-0.6	5.6E-07
	Chromosome	8	0.1	0.3	2.7E-06
	Chromosome	14	0.1	0.3	1.5E-05
	Chromosome	18	0.0	-0.4	2.9E-05
	Chromosome	7	-0.1	0.2	8.5E-05
	Chromosome	11	0.0	0.2	1.9E-03
	Chromosome	22	0.1	0.2	1.2E-02
	Chromosome	12	-0.1	0.1	1.3E-02
	Chromosome	17	0.1	0.0	2.1E-02
	Corum	55S ribosome, mitochondrial	0.5	0.1	6.1E-04
	Corum	39S ribosomal subunit, mitochondrial	0.6	0.1	3.6E-03
	Corum	Ribosome, cytoplasmic	-0.4	-0.1	4.2E-03
	GOBP	cell communication	-0.2	0.0	3.7E-03
	GOBP	cell adhesion	-0.4	0.1	4.1E-03
	GOBP	signal transduction	-0.2	0.0	5.3E-03
	GOBP	biological adhesion	-0.4	0.1	1.1E-02
	GOBP	regulation of actin polymerization or depolymerization	-0.6	0.0	1.5E-02
	GOBP	cell motion	-0.4	0.2	2.2E-02
	GOBP	translational elongation	-0.4	-0.1	3.8E-02
	GOCC	mitochondrial part	0.4	0.0	0.0E+00
	GOCC	mitochondrial inner membrane	0.5	-0.1	3.9E-08
	GOCC	organelle inner membrane	0.5	-0.1	4.3E-07
	GOCC	cytoskeleton	-0.4	0.0	1.2E-06
	GOCC	cytoplasm	-0.2	0.0	3.0E-06
	GOCC	mitochondrion	0.3	0.0	3.3E-06
	GOCC	mitochondrial membrane	0.4	0.0	5.5E-06
	GOCC	cytosol	-0.2	0.0	1.1E-05
	GOCC	mitochondrial matrix	0.4	0.0	6.2E-05
	GOCC	integral to membrane	0.2	0.0	1.5E-04
	GOCC	intrinsic to membrane	0.2	0.0	3.6E-04
	GOCC	cytoskeletal part	-0.3	0.0	2.3E-03
	GOCC	organelle membrane	0.1	0.0	1.2E-02
	GOCC	cell-substrate adherens junction	-0.5	0.1	1.4E-02

	GOCC	cytosolic part	-0.4	-0.1	1.7E-02
	GOCC	cell-substrate junction	-0.5	0.1	1.7E-02
	GOCC	nucleoid	0.5	-0.1	2.1E-02
	GOCC	cell projection	-0.3	0.0	2.1E-02
	GOCC	adherens junction	-0.4	0.0	2.2E-02
	GOCC	mitochondrial nucleoid	0.5	-0.1	3.6E-02
	GOCC	membrane part	0.1	0.0	3.7E-02
	GOCC	focal adhesion	-0.5	0.1	3.7E-02
	GOMF	cytoskeletal protein binding	-0.3	0.0	1.8E-04
	GOMF	actin binding	-0.4	0.0	2.7E-04
	GOMF	cation transmembrane transporter activity	0.4	0.0	6.0E-03
	GOMF	ion transmembrane transporter activity	0.4	-0.1	6.7E-03
	KEGG	Oxidative phosphorylation	0.5	0.0	4.5E-05
	KEGG	Regulation of actin cytoskeleton	-0.5	0.0	4.9E-05
	KEGG	Focal adhesion	-0.5	0.0	7.2E-05
	KEGG	Parkinson's disease	0.4	0.0	1.3E-03
	KEGG	Ribosome	-0.4	0.0	1.4E-03
	KEGG	Huntington's disease	0.3	0.0	7.9E-03
	KEGG	Alzheimer's disease	0.3	0.0	3.3E-02
	KEGG	Leukocyte transendothelial migration	-0.4	-0.1	3.5E-02
	KEGG	MAPK signaling pathway	-0.3	0.0	3.7E-02
	KEGG	Pathways in cancer	-0.3	0.0	3.9E-02
	KEGG	Cardiac muscle contraction	0.5	0.1	4.3E-02
HCC1143	Chromosome	22	0.3	0.7	0.0E+00
	Chromosome	4	-0.2	-0.5	4.1E-14
	Chromosome	X	0.0	-0.4	2.3E-07
	Chromosome	8	0.1	0.4	4.8E-07
	Chromosome	3	0.0	0.3	5.5E-07
	Chromosome	16	-0.1	-0.3	1.8E-06
	Chromosome	20	0.2	0.3	2.4E-06
	Chromosome	11	-0.1	-0.3	2.5E-06
	Chromosome	7	-0.1	-0.2	7.9E-05
	Chromosome	14	0.1	0.3	1.5E-04
	Chromosome	18	0.1	0.4	1.7E-04
	Chromosome	21	0.3	0.4	1.8E-04
	Chromosome	19	0.2	0.1	4.5E-04
	Chromosome	5	-0.1	-0.2	2.8E-03
	Chromosome	15	-0.1	-0.2	1.9E-02
	Chromosome	17	0.0	0.1	2.8E-02
	Corum	Spliceosome	0.4	0.1	4.6E-05
	Corum	ALL-1 supercomplex	0.7	-0.2	3.3E-03
	Corum	C complex spliceosome	0.4	0.1	1.0E-02
	Corum	Respiratory chain complex I (holoenzyme), mitochondrial	-0.7	-0.1	1.1E-02
	Corum	Ribosome, cytoplasmic	-0.4	0.0	1.4E-02
	Corum	anti-HDAC2 complex	0.7	-0.2	2.5E-02
	Corum	SNF2h-cohesin-NuRD complex	0.7	-0.2	2.8E-02
	GOBP	nucleobase, nucleoside, nucleotide and nucleic acid metabolic process	0.2	0.0	0.0E+00
	GOBP	transcription	0.3	0.0	5.2E-08
	GOBP	nitrogen compound metabolic process	0.2	0.0	6.7E-08

	GOBP	chromatin organization	0.4	-0.1	3.0E-06
	GOBP	DNA metabolic process	0.3	0.0	5.1E-06
	GOBP	regulation of transcription, DNA-dependent	0.2	0.0	3.2E-05
	GOBP	DNA replication	0.4	-0.1	1.4E-03
	GOBP	RNA splicing, via transesterification reactions with bulged adenosine as nucleophile	0.4	0.1	2.4E-03
	GOBP	RNA splicing, via transesterification reactions	0.4	0.1	2.6E-03
	GOBP	nuclear mRNA splicing, via spliceosome	0.4	0.1	2.9E-03
	GOBP	nuclear division	0.4	0.0	3.7E-03
	GOBP	transport	-0.1	0.0	8.0E-03
	GOBP	mitochondrial electron transport, NADH to ubiquinone	-0.7	-0.1	1.4E-02
	GOBP	lipid metabolic process	-0.2	0.0	2.2E-02
	GOBP	mitosis	0.4	-0.1	2.3E-02
	GOCC	nuclear part	0.3	0.0	0.0E+00
	GOCC	nucleus	0.2	0.0	3.6E-08
	GOCC	chromosomal part	0.4	-0.2	1.7E-05
	GOCC	chromatin remodeling complex	0.5	-0.4	4.2E-04
	GOCC	nucleoplasm	0.3	0.0	4.7E-04
	GOCC	spliceosomal complex	0.4	0.1	7.1E-04
	GOCC	nucleoplasm part	0.3	0.0	2.9E-03
	GOCC	mitochondrial respiratory chain complex I	-0.7	-0.1	3.9E-03
	GOCC	NADH dehydrogenase complex	-0.7	-0.1	6.1E-03
	GOCC	membrane	-0.1	0.0	1.5E-02
	GOCC	ribosome	-0.3	0.0	1.6E-02
	GOCC	chromatin	0.4	-0.2	1.9E-02
	GOCC	nuclear body	0.3	0.0	2.0E-02
	GOCC	ribosomal subunit	-0.3	0.0	2.4E-02
	GOCC	nucleolus	0.2	0.0	5.0E-02
	GOMF	DNA binding	0.3	-0.1	6.8E-11
	GOMF	nucleic acid binding	0.2	0.0	8.1E-11
	GOMF	structural molecule activity	-0.2	0.0	1.7E-03
	GOMF	structural constituent of ribosome	-0.3	0.0	6.1E-03
	GOMF	NADH dehydrogenase activity	-0.7	-0.1	6.4E-03
	GOMF	NADH dehydrogenase (ubiquinone) activity	-0.7	-0.1	7.7E-03
	GOMF	transcription factor activity	0.3	-0.1	3.7E-02
	GOMF	chromatin binding	0.5	-0.1	3.8E-02
	GOMF	oxidoreductase activity, acting on NADH or NADPH	-0.5	0.0	4.5E-02
	KEGG	DNA replication	0.7	0.1	2.3E-03
	KEGG	Ribosome	-0.4	0.0	4.5E-03
	KEGG	Focal adhesion	-0.2	0.2	1.3E-02
