# Supplementary material for: Proteomic Changes Resulting from Gene Copy Number Variations in Cancer Cells
Source: PLoS Genet. 2010 Sep 2;6(9):e1001090. doi: 10.1371/journal.pgen.1001090 (PMC2932691; doi:10.1371/journal.pgen.1001090)
Supplement: Table S2 — Cancer-associated genes that have a change in gene copy number. (0.08 MB PDF) [file pgen.1001090.s004.pdf]

Table S2: Cancer associated genes that have a change in gene copy number. Proteomic and genomic data was compared to the cancer gene census" published by the welcome trust Sanger institute. Table indicates deleted and amplified genes that positively correlate with the protein level.

Cell	Gene name	Log 2 Ratio proteome	Log2 Ratio genome	Cancer type	Cancer molecular genetics	Mutation type
HCC2218	CDH1	-3.0	-3.1	lobular breast, gastric	Rec	Mis, N, F, S
	MAP2K4	-1.1	-1.0	pancreatic, breast, colorectal	Rec	D, Mis, N
	TAF15	-0.6	-1.0	chondrosarcomas, ALL	Dom	T
	LASP1	-1.6	-0.9	AML	Dom	T
	HIP1	-1.0	-0.8	CMML	Dom	T
	CBFB	-1.8	-0.7	AML	Dom	T
	HRPT2	0.5	0.6	parathyroid adenoma	Rec	Mis, N, F
	TOP1	1.8	0.6	AML	Dom	T
	GOLGA5	1.5	0.7	papillary thyroid	Dom	T
	PRKAR1A	1.0	1.0	papillary thyroid	Dom, Rec	T, Mis, N, F, S
	COX6C	3.5	1.1	uterine leiomyoma	Dom	T
	FH	1.8	1.1		Rec	Mis, N, F
	TCEA1	1.7	1.1	salivary adenoma	Dom	T
	ERBB2	5.8	2.3	breast, ovarian	Dom	A, Mis, O
HCC1143	LASP1	-1.1	-0.8	AML	Dom	T
	SDHB	-1.3	-0.8		Rec	Mis, N, F
	ERCC4	-0.5	-0.7		Rec	Mis, N, F
	LPP	0.7	0.5	lipoma, leukemia	Dom	T
	SS18	0.9	0.5	synovial sarcoma	Dom	T
	TIF1	1.5	0.5	APL	Dom	T
	CEP1	2.0	0.5	MPD, NHL	Dom	T
	SMARCB1	1.6	0.6	malignant rhabdoid	Rec	D, N, F, S
	NF2	3.3	0.6	meningioma, acoustic neuroma	Rec	D, Mis, N, F, S, O
	COX6C	0.9	0.6	uterine leiomyoma	Dom	T
	TFG	0.8	0.6	papillary thyroid, ALCL	Dom	T
	D10S170	0.5	0.6	papillary thyroid, CML	Dom	T
	TAF15	0.9	0.6	extraskeletal myxoid chondrosarcomas, ALL	Dom	T
	CDK4	0.5	0.7		Dom	Mis
	EP300	1.4	0.7	colorectal, breast, pancreatic, AML	Rec	T
	TPM3	0.6	0.7	papillary thyroid, ALCL	Dom	T
	ZNF278	3.1	0.7	Ewing sarcoma	Dom	T
	MSN	0.7	0.8	ALCL	Dom	T
	FANCD2	3.5	0.8		Rec	D, Mis, N, F
	SSH3BP1	0.8	0.8	AML	Dom	T
	FIP1L1	1.5	0.9	idiopathic hypereosinophilic syndrome	Dom	T
	EWSR1	0.8	0.9	Ewing sarcoma, desmoplastic small round cell tumor , ALL	Dom	T
	AKT1	1.1	1.5	breast, colorectal, ovarian, NSCLC	Dom	Mis
	CCND1	2.2	2.4	CLL, B-ALL, breast	Dom	T
	BRD4	2.1	2.5	lethal midline carcinoma of young people	Dom	T
